# The associations between childhood vaccine history and COVID-19 vaccine uptake among older adults in 28 high-income countries

**DOI:** 10.21203/rs.3.rs-5496025/v1

**Published:** 2025-11-20

**Authors:** Xu Zong, Karri Silventoinen

**Affiliations:** 1. Helsinki Institute for Demography and Population Health, Faculty of Social Sciences, University of Helsinki, Helsinki, Finland; 2. Max Planck - University of Helsinki Center for Social Inequalities in Population Health, Helsinki, Finland

**Keywords:** Vaccine hesitancy, Childhood vaccination, COVID-19 vaccine uptake, SHARE, Life course, Vaccine behaviors

## Abstract

**Background:**

Increasing vaccination uptake is critical for population health. However, little is known about the how childhood vaccination affects vaccination intake in adulthood. This study aims to examine the long-term associations between childhood vaccination history and COVID-19 vaccine uptake among older adults.

**Method:**

The study utilized data from the Survey of Health, Aging and Retirement in Europe, which included information on demographic variables, childhood vaccination, and COVID-19 vaccine uptake. Daily COVID-19 stringency data from Our World in Data was also incorporated into the study. The study cohort consisted of 48,963 participants from 27 European countries and Israel, of whom 81% (39,653) were vaccinated against COVID-19. A lasso regression machine learning approach was utilized to identify key confounders from a pool of 19 potential confounders. Logistic regression was then used to analyze the association between childhood vaccination and COVID −19 vaccine uptake in later life.

**Result:**

Childhood vaccination was found to be associated with COVID-19 vaccine uptake among middle-aged and older adults (OR = 1.66, 95% CI = [1.42, 1.94], p < 0.001) after adjusting for 16 demographics, mitigation behavior and COVID-19-related variables. Older age significantly enhanced this association.

**Conclusions:**

This study is the first to demonstrate a significant association between childhood vaccine history and adult vaccine behavior. This highlights the importance of childhood vaccination status in promoting COVID-19 vaccination among older adults. These findings support policies that emphasize the long-term value of childhood vaccination.

## 1. Background

The COVID-19 pandemic has had a significant impact on global health [1, 2]. Older adults were particularly vulnerable to the disease [3, 4], facing severe health outcomes such as a higher risk of infection [5], increased mortality rates [6], and a high prevalence of mental health problems [7, 8]. COVID-19 vaccination has been a critical tool in protecting older adults from the infection with strong evidence for reducing the risk of infection and lowering mortality rates after infection [9]. Understanding the determinants of COVID-19 vaccine uptake is essential for encouraging older adults to get vaccinated. Previous studies have explored a variety of factors that influence COVID-19 vaccine hesitancy and uptake, including age [10], gender [11], education level [10–12], marital status [12], health literacy [13], health status [14, 15], and risk perceptions [16]. However, these studies have primarily focused on factors in later life, leaving a gap in knowledge about how early-life circumstances, particularly childhood vaccination, might impact vaccine uptake in older age.

Childhood vaccination is a crucial early-life factor that has a significant impact on later-life health outcomes [17–19]. A historical analysis from the Netherlands showed that childhood vaccination reduced child mortality rates in the 20th century [20], and a Danish case-cohort study confirmed its association with improved long-term survival [21]. In addition to the health benefits, childhood vaccination has also been linked to enhanced cognitive performance [22, 23] and increased educational attainment in adulthood [24, 25]. Previous research has shown that childhood circumstances can influence further health care utilization [26, 27] and the willingness to receive the COVID-19 vaccine in early adulthood [28].

Despite extensive previous research, no study has yet examined the association between childhood vaccination and COVID-19 vaccine uptake in old age. Investigating this association poses challenges, primarily due to the lack of comprehensive data on the history of vaccination during childhood and vaccination uptake in older age. To address this gap, our study aims to explore the relationship between childhood vaccination and COVID-19 vaccine uptake among middle-aged and older adults in 28 high-income countries. By merging data on childhood vaccination with COVID-19 vaccine uptake during the pandemic, this study contributes to the literature on childhood vaccines and COVID-19 vaccine uptake. Additionally, this study examines the potential moderating effect of age on this relationship.

## The Potential Mechanisms

Childhood vaccination can promote later-life COVID-19 vaccine uptake through several mechanisms. First, childhood vaccination can shape long-term health behaviors by normalizing preventive health care and promoting pro-vaccine attitudes. Those who received vaccines in early life may be more likely to perceive vaccines as effective and safe, and therefore be more inclined to receive them later in life. Second, early vaccination experiences can promote trust in medical systems and public health institutions throughout life. This can increase COVID-19 vaccine uptake, as trust in public health institutions has been found to be a significant predictor of COVID-19 vaccination [29]. Third, research has shown that childhood factors can influence health literacy in adulthood [30, 31]. Individuals with a history of childhood vaccination may have higher health literacy, which has been linked to higher rates of COVID-19 vaccine acceptance [13]. Based on this body of literature, we propose Hypothesis 1: childhood vaccine history can increase the likelihood of COVID-19 vaccine uptake in older adults.

## Moderating Effect of Age

Older individuals may demonstrate stronger behavioral connections to early-life health experiences, including childhood vaccination. According to the theory of cumulative advantage/disadvantage [32, 33], initial benefits, such as early exposure to preventive health care, can accumulate over the life course reinforcing pro-vaccination attitudes and trust in the health care system. As individuals age, these accumulated advantages have a greater impact on later-life health decisions. Conversely, those without childhood vaccination experiences may accumulate disadvantages in the form of weaker vaccine confidence or limited engagement with preventive care. Additionally, older adults tend to perceive themselves as being at higher risk for severe COVID-19 outcomes, which may motivate them to rely on and act upon prior positive vaccination experiences [34, 35]. Therefore, we propose Hypothesis 2: Age can moderate the association between childhood vaccine history and COVID-19 vaccine uptake.

## 2. Methods

### 2.1. Participants

This study used multinational data from the Survey of Health, Aging and Retirement in Europe (SHARE), a multidisciplinary research infrastructure spanning 28 European countries and Israel. SHARE started in 2004 with nationally representative participants from 11 European countries. The most recent data collection was the wave 9, conducted in 2022, providing information on the health status, family structure, social networks, economic situation, and life history of individuals aged 50 and over [36]. During the COVID-19 pandemic, SHARE Corona Survey 1 and SHARE Corona Survey 2 were conducted in 2020 and 2021, respectively, covering major life domains of the participants, such as health and health behavior, mental health, infections and healthcare, changes in work and economic situation, and social networks [37]. SHARE waves 1 to 4 received the ethical approval from the Ethics Committee of the University of Mannheim, and subsequent waves were approved by the Ethics Council of the Max Planck Society. In addition to using SHARE data, COVID-19 lockdown stringency was measured by calculating the 30-day average of the COVID-19 stringency index prior to each participant’s interview date. This calculation was based on daily COVID-19 stringency data obtained from Our World in Data [38].

Previous studies have utilized SHARE data to examine the factors influencing the willingness of older adults to receive the COVID-19 vaccine, including prayer frequency [39], family size [40], prior influenza vaccination [41] and social capital [42]. However, these studies have mainly focused on factors in later life. No previous studies have utilized a life-course perspective to investigate the early-life determinants of COVID-19 vaccine uptake during the pandemic.

### 2.2. Measures

The dependent variable in this study was the uptake of the COVID-19 vaccine. In the SHARE Corona Survey 2, participants were asked: “Have you been vaccinated against COVID-19?” with the response options “Yes” and “No”. Using these responses, a binary variable was created: 1 indicated having received the COVID-19 vaccine, while 0 indicated not receiving the vaccine.

Childhood vaccination was the key independent variable. In SHARE wave 7, participants were asked: ‘During your childhood, that is, before you turned 16, have you received any vaccinations?’ with response options ‘Yes’ and ‘No’. A binary variable based on these responses was then created: 1 indicated having a history of vaccination during childhood, while 0 indicated not having a history of vaccination during childhood.

To consider potential factors that may affect COVID-19 vaccine uptake, 19 confounders across three domains were examined, based on previous research [39, 40, 42, 43]. The first domain focused on demographics, including gender (men, women), marital status (married or partnered, not married or partnered), age (years), years of schooling (years), living in institutions (yes, no), living in rural areas (yes, no), geographic regions (Northern Europe, including Sweden, Denmark and Finland; Southern Europe, including Spain, Italy, Greece, Cyprus, Malta, Portugal; Eastern Europe, including Slovenia, Estonia, Lithuania, Bulgaria, Latvia, Romania, Poland, Hungary, Slovakia, Czech Republic, Croatia; Western Europe, including Netherlands, France, Belgium, Ireland, Luxembourg, Germany and Switzerland; Israel), self-rated health status (excellent, very good, good, fair, poor), frequency of contacting children online (daily, several times a week, about once a week, less often, never), and frequency of seeing children in person (daily, several times a week, about once a week, less often, never). The second domain included mitigation behavior confounders, such as adherence to social distancing guidelines (never, sometimes, often, always), receiving flu vaccination (yes, no), and taking medication for COVID prevention (yes, no). The third domain comprises COVID-19-related factors, including COVID-19 lockdown stringency. This was calculated as the 30-day average COVID-19 stringency index prior to each participant’s interview date, using daily data from Our World in Data. The index reflects the average score of nine metrics: school closures, workplace closures, cancellation of public events, restrictions on public gatherings, public transport closures, stay-at-home orders, public information campaigns, restrictions on internal movements, and international travel controls. A higher index indicates a stricter COVID-19 lockdown. This domain also includes factors like testing positive for COVID-19 (yes, no), ever experienced any COVID symptoms (yes, no), delaying medical care (yes, no), losing a close person to COVID (yes, no), and being hospitalized for COVID (yes, no).

### 2.3 Method

Lasso (Least absolute shrinkage and selection operator) regression, logistic regression, and interaction item analysis were utilized. Lasso is a machine learning approach that is good at selecting relevant independent variables from a large set of potential predictors [44]. It utilizes a penalty function to shrink the coefficients of confounders, effectively narrowing the coefficients of insignificant confounders to zero. Lasso regression identifies the key confounders based on the optimal lambda value (lambda.1se) by using 10-fold cross-validation. Logistic regression is an effective approach to examine the association between the binary dependent variable and key independent variables. Interaction item analysis is used to explore how a third variable moderates the relationship between two other variables.

We initially utilized lasso regression to identify key confounders from the three domains of potential confounders: demographics, mitigation behavior, and COVID-19 related confounders. After applying lasso regression, 16 of the original 19 confounders were retained, including marital status, living in institutions, living in rural areas, self-rated health status, frequency of seeing children in person, age, years of schooling, adherence to social distancing guidelines, taking medication for COVID-19 prevention, receiving flu vaccination, testing positive for COVID-19, ever experienced any COVID symptoms, delaying medical care, COVID-19 lockdown stringency, losing a close person to COVID, and being hospitalized for COVID. These key confounders were then incorporated into the logistic regression models to assess the association between childhood vaccination and COVID-19 vaccine uptake among middle-aged and older participants, while adjusting for the identified confounders. Finally, interaction terms were added to the logistic regression models to investigate the moderating effects of age. The analyses were performed using R (version 4.3.1) and Stata (version 18.0).

## 3. Results

### 3.1. Descriptive Analysis

**Figure 1.**
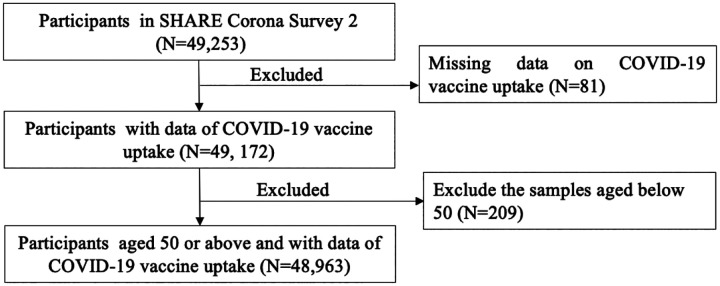
Flow chart of participants selecting

There were 49,253 participants in the SHARE Corona Survey 2. After excluding participants under 50 years old and those with missing values on COVID-19 vaccine uptake, 48,963 participants were included in the analyses (Figure 1). The random forest model was used to impute the missing information of independent variables, due to its advantage in handling data with non-linear relationships and interactions [45, 46].

The average age of participants was 71 years old, with 58% (28,448) being women, and 81% (39,653) having received the COVID-19 vaccine. Differences in COVID-19 vaccine uptake across categorical sociodemographic variables were assessed using the chi-square tests, while t-tests were used for continuous variables such as age and years of schooling. Those who had received the COVID-19 vaccination were more likely to have been vaccinated in childhood, be male, be married or partnered, and have a higher education level (Table A1 in the Appendix).

### 3.2. Empirical Results

**Figure 2.**
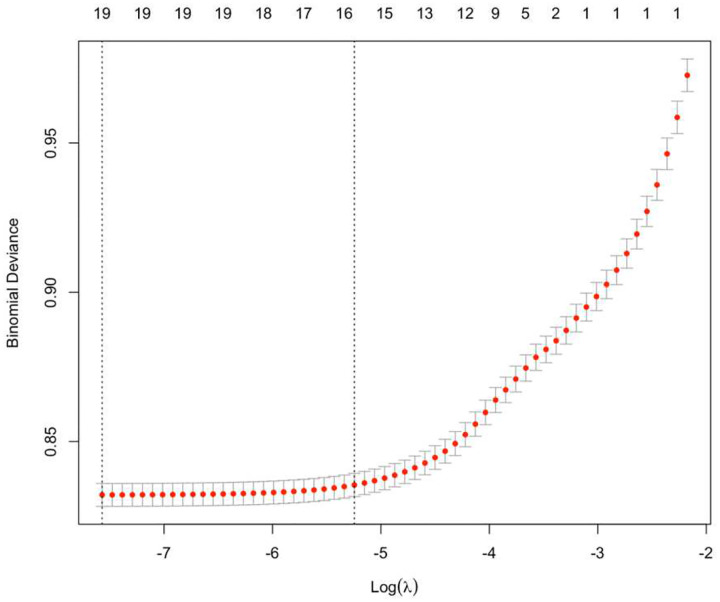
Cross-validation results for optimal lambda selection

We identified 16 confounders with non-zero coefficients at the optimal lambda value (λ= 0,005, corresponding to lambda.1se) (Figure 2) and included them in logistic regression models. Logistic regression models were established to examine the association between childhood vaccination and COVID-19 vaccine uptake in later life. The results from Model 1 to Model 3 are presented in Table A2 in the Appendix showing the stepwise inclusion of confounding variables and interaction terms.

Model 1 serves as the baseline model, including only childhood vaccination variable. The results indicate a significant association between childhood vaccination and COVID-19 vaccine uptake in later life (OR = 1.79, 95% CI = [1.56, 2.05], p < 0.001). This suggests that individuals with a history of childhood vaccination were more likely to receive the COVID-19 vaccine

Next, confounding variables from the domains of demographics, mitigation behaviors and COVID-19-related factors were included in the analyses (Model 2). The results indicate that childhood vaccination remains positively associated with COVID-19 vaccine uptake, although the effect size decreased (OR = 1.66, 95% CI = [1.42, 1.94], p < 0.001). This means that those who had childhood vaccination were still 67% more likely to receive the COVID-19 vaccine after adjusting for these covariates. Additionally, all confounding variables were significantly associated with COVID-19 vaccine uptake. In particular, higher schooling years, older ages, being married or partnered, living in institutions, seeing children in person, maintaining social distance, receiving flu vaccination, high levels of COVID-19 lockdown stringency, and forgoing medical care were all significantly associated with an increased likelihood of COVID-19 vaccine uptake. Conversely, factors such as living in rural areas, poorer self-rated health status, ever tested positive for COVID-19, ever experienced any COVID symptoms, and ever taking medication for COVID prevention were significantly associated with a decreased likelihood of receiving the COVID-19 vaccine.

**Figure 3.**
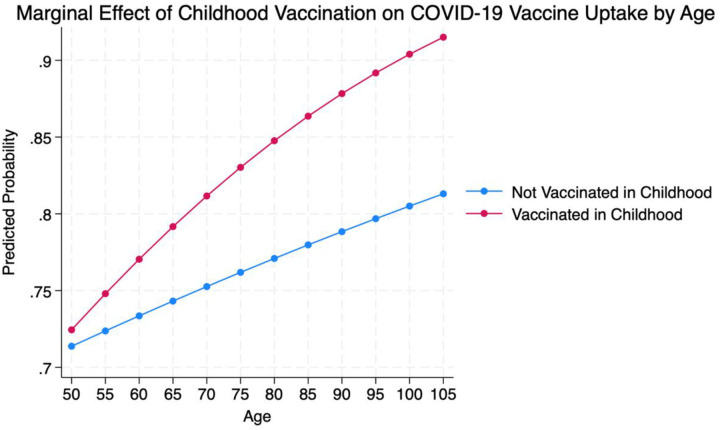
Interaction between childhood vaccination and age in predicting COVID-19 vaccine uptake: Predicted probabilities by age group Note: Predicted probabilities are shown across ages 50 to 105, separately for individuals with and without a history of childhood vaccination. The increasing gap between the two lines suggests that the effect of childhood vaccination on later-life vaccine uptake becomes more pronounced at older ages.

Finally, an interaction term (childhood vaccination × age) was included in the model to investigate if the association between childhood vaccination and COVID-19 vaccine uptake is influenced by age (Model 3). This interaction term was significant (OR = 1.02, 95% CI = [1.00, 1.03], p < 0.05), indicating that the association between childhood vaccination and COVID-19 vaccine uptake in late life is more pronounced among older participants. The predicted probability of COVID-19 vaccine uptake increased with age for groups with or without a childhood vaccination history (Figure 3). However, the difference in uptake between individuals with and without a childhood vaccination history was more pronounced at older ages. This supports the presence of a significant interaction effect, suggesting that early-life vaccination may have a stronger influence on vaccine behavior among the oldest age groups.

### 3.3. Sensitive Analysis

As an alternative to the random forest model, we used mode imputation for categorical variables and median imputation for continuous variables to address missing data in childhood vaccination history and 16 important confounders (Table A3 in the Appendix). These methods have been extensively used in previous studies and have been shown to be valid for imputing values [47–49]. The associations between childhood vaccine history and COVID-19 vaccine uptake among older adults in 28 high-income countries remained robust when re-estimated using the imputed data. The effect size is 1.66 (95% CI: [1.43, 1.94], p < 0.001), consistent with the results from the random forest imputation.

Additionally, instead of selecting important confounders through lasso regression, we re-estimated the model using the full set of control variables (see Table A3 in the Appendix). The statistical significance of childhood vaccine history remained consistent, although the estimated effect size slightly declined from 1.66 to 1.58.

## 4. Discussion

To the best of our knowledge, this is the first study investigating the association between childhood vaccination and adult vaccine behavior from a life-course perspective. The main findings demonstrate that individuals who received vaccinations during childhood were more likely to receive the COVID-19 vaccine in later life. Moreover, the association was modified by age, with older age groups showing a stronger relationship between childhood vaccination and COVID-19 vaccine uptake. This study also demonstrated significant associations between a range of confounders and COVID-19 vaccine uptake.

Previous studies have revealed the long-term effects of childhood vaccination, such as decreased mortality [20], better long-term survival [21], better cognitive abilities [22, 23], and higher educational attainment [24, 25]. These findings support the association between childhood vaccination and positive health outcomes throughout life. Similarly, our study confirmed a relationship between childhood vaccination and positive health behaviors, specifically COVID-19 vaccine uptake. Individuals aged 50 years and older who had childhood vaccination are 66% more likely to receive the COVID-19 vaccine compared to those who did not (95% CI = [1.43, 1.94], p < 0.001). This finding highlights the importance of the life-course perspective when studying the determinants of vaccination, as the benefits of childhood vaccination appear to persist into old age.

There are several potential explanations for the higher prevalence of COVID-19 vaccine uptake among older adults with a history of childhood vaccination. First, these individuals may have improved health literacy due to increased exposure to information about vaccines and health throughout their lives. A higher level of health literacy may increase the acceptance of the COVID-19 vaccine [50]. Additionally, vaccination as a health behavior established in early life may persist into adulthood, as individuals may have recognized the benefits from childhood vaccinations. Finally, receiving vaccinations during childhood may enhance trust in the medical system, which is critical for encouraging COVID-19 vaccine uptake.

Our findings indicate that the associations between childhood vaccination and COVID-19 vaccination uptake later in life are modified by age, with older individuals showing a stronger association. This aligns with previous studies that have shown a link between age and COVID-19 vaccine uptake [51]. The impact of age on this relationship can be understood through the cumulative advantage/disadvantage theory [32, 33]. This theory suggests that early-life exposures and resources, like childhood vaccination, can lead to lasting behavioral advantages that accumulate over time.

Older adults who were vaccinated as children may have developed stronger health-seeking behaviors and greater trust in public health systems, which may become more stable and influential as they age. Older adults can also perceive themselves to be at a higher risk of infection, leading them to be more motivated to receive the vaccine. Additionally, we found significant associations between various confounding variables and COVID-19 vaccine uptake. For example, demographic factors, such as age, marital status and education, were correlated with COVID-19 vaccine uptake. This aligns with previous research that emphasizes the role of socioeconomic factors in influencing vaccine acceptance [43, 51, 52].

The association between childhood vaccination and vaccine behavior in later life highlights the importance of adopting a life-course perspective in vaccination promotion strategies. Public health efforts may benefit from targeted outreach to individuals who have not received vaccinations in childhood. Furthermore, maintaining comprehensive lifelong vaccination records and incorporating vaccine education into early health curricula could enhance vaccine uptake and strengthen public trust in future immunization campaigns. These efforts should be supported by ethically sensitive and culturally appropriate communication strategies. Additionally, our findings underline the potential long-term benefits of childhood vaccination, not only for immediate disease prevention but also in fostering positive vaccine-related behaviors in later life. In light of increasing anti-vaccination sentiments targeting childhood immunization, reinforcing early-life vaccine programs and public education may serve as a proactive strategy to build vaccine confidence and health system resilience for future public health crises. Moreover, the moderating effect of age between childhood vaccination and COVID-19 vaccine uptake highlights the importance of considering age-specific strategies when promoting vaccination later in life.

There are some strengths in our study. First, this study utilized a large, nationally representative sample of adults aged 50 years and over from 28 high-income countries. Second, this study was the first to identify the long-term effect of childhood vaccination on COVID-19 vaccine uptake in late life, thereby expanding the literature on the enduring health effects of childhood vaccination. Lastly, this study used data on COVID-19 vaccine uptake instead of data on the willingness to receive the vaccine, which may better reflect real-world behavior. However, there are also limitations in this study. First, our study was focused on high-income countries, and hence the findings may not be generalizable to low- and middle-income countries. Second, information on childhood vaccination was based on participants’ recall, and COVID-19 vaccine uptake was self-reported, which could introduce bias. Third, there is substantial variation in COVID-19 vaccination policies and uptake across European countries [42, 53], with differences partly explained by unobserved regional factors. While our study focuses on individual-level associations, future research should further explore how macro-level differences such as public health systems and vaccine delivery infrastructure across national contexts influence vaccine behaviors and interact with childhood vaccination. Additionally, it is important to acknowledge that childhood vaccination uptake in the study population may have been shaped by institutional and policy contexts. In many European countries, childhood vaccination was mandatory as part of national public health strategies [54]. Consequently, variation in childhood vaccination history was limited, with only a small percentage of respondents reporting no vaccination. Despite this, our findings indicate a statistically significant association between childhood vaccination and COVID-19 vaccine uptake in later life. This suggests that early-life exposure to vaccination, whether mandated or voluntary, may have enduring effects on vaccine acceptance. Future studies could further investigate how the nature and strength of childhood vaccination policies (e.g., compulsory vs. voluntary) influence long-term vaccine-related behaviors. Finally, as a cross-sectional study, this study examined the association between childhood vaccination and COVID-19 vaccine uptake in late life, but it did not explore the underlying mechanism or establish causality. Future research should include more representative datasets from developing countries and explore the potential mechanisms.

## 5. Conclusion

We demonstrated that adults who received vaccinations during childhood are more likely to receive the COVID-19 vaccine, and this association was stronger in older adults compared to middle-aged adults. These findings highlight the potential long-term benefits of childhood vaccination in encouraging positive health behaviors throughout life. This suggests that early vaccination efforts not only protect against immediate health risks but also foster a stronger commitment to vaccination in adulthood. Additionally, these finding provide insights for health care professionals seeking to promote COVID-19 vaccine acceptance. Given that individuals without a history of childhood vaccination are less inclined to receive the COVID-19 vaccine, targeted strategies should be implemented for this group. These findings support policies emphasizing the enduring benefits of childhood vaccines.

## Data Availability

The original data presented in the study are openly available on the website https://share-eric.eu/ and https://ourworldindata.org/.

## Appendix

**Table A1.** Descriptive characteristics by COVID-19 vaccine uptake or not

Variable	Total participants	COVID-19 vaccine uptake	No COVID-19 vaccine uptake	p-value
**Mean age (SD)**	71 (9)	71 (9)	69 (10)	< 0.001
**Mean schooling years (SD)**	11 (4)	11 (4)	10 (4)	< 0.001
**Gender (%)**				< 0.001
Male	20,515 (42)	16,928 (83)	3,587 (17)	
Female	28,448 (58)	22,725 (80)	5,723 (20)	
**Married or partnered (%)**				< 0.001
Yes	33,854 (69)	28,053 (83)	5,801 (17)	
No	15,109 (31)	11,600 (77)	3,509 (23)	
**Geographic regions (%)**				< 0.001
Northen Europe	3,859 (8)	3,733 (97)	126 (3)	
Southern Europe	11,015 (23)	9,674 (88)	1,341 (12)	
Eastern Europe	19,870 (41)	13,398 (67)	6,472 (33)	
Western Europe	12,941(26)	11,649(90)	1,292(10)	
Israel	1,278 (3)	1,199 (94)	79 (6)	
**Childhood vaccination (%)**				< 0.001
Yes	47,969 (97)	38,950 (81)	9,019 (19)	
No	994 (3)	703 (71)	291 (29)	

**Table A2.** Results of logistic regression models (n = 48,963)

Variables	Model 1	Model 2	Model 3
	OR (95% CI)	OR (95% CI)	OR (95% CI)
Childhood vaccination (Ref. = No)	1.79*** (1.56, 2.05)	1.66*** (1.42, 1.94)	0.45 (0.14, 1.48)
Schooling years		1.06*** (1.05,1.06)	1.06*** (1.05, 1.06)
Age		1.03*** (1.03,1.03)	1.01 (1.00, 1.03)
Being married or partnered (Ref. = No)		1.52*** (1.44,1.60)	1.52*** (1.44,1.60)
Living in rural areas (Ref. = No)		0.80*** (0.76, 0.84)	0.80*** (0.76, 0.84)
Living in institutions (Ref. = No)		2.11*** (1.50, 2.98)	2.12*** (1.50, 2.98)
Self-rated health status (Ref. = Excellent)			
Very good		0.88 (0.77, 1.02)	0.88 (0.77, 1.02)
Good		0.76*** (0.67, 0.87)	0.76*** (0.67, 0.87)
Fair		0.65*** (0.57, 0.74)	0.65*** (0.57, 0.74)
Poor		0.41*** (0.35, 0.47)	0.41*** (0.35, 0.47)
Seeing children in person (Ref. = Daily)			
Several times a week		1.34*** (1.24, 1.43)	1.34***(1.24, 1.44)
About once a week		1.47***(1.36, 1.58)	1.47***(1.36, 1.58)
Less often		1.27***(1.19, 1.36)	1.27***(1.18, 1.36)
Never		1.00 (0.89, 1.11)	1.00 (0.89, 1.11)
Keeping social distance (Ref. = Never)			
Sometimes		1.22* (1.03, 1.44)	1.22* (1.03, 1.44)
Often		1.48*** (1.27, 1.72)	1.48*** (1.27, 1.72)
Always		1.69*** (1.46, 1.95)	1.69*** (1.46, 1.95)
Taking medication for COVID prevention (Ref. = No)		0.83*** (0.77, 0.89)	0.83*** (0.77, 0.89)
Receiving flu vaccination (Ref. = No)		6.49*** (6.03, 6.99)	6.49*** (6.02, 6.99)
Ever tested positive for COVID-19 (Ref. = No)		0.70*** (0.62, 0.80)	0.70*** (0.62, 0.80)
Ever experienced any COVID symptoms (Ref. = No)		0.58*** (0.51, 0.66)	0.58*** (0.51, 0.66)
Forgoing medical care (Ref. = No)		1.48*** (1.39, 1.58)	1.48*** (1.39, 1.59)
COVID-19 lockdown stringency		1.02*** (1.02, 1.03)	1.02***(1.02, 1.03)
Losing a close person to COVID		1.53*** (1.39, 1.68)	1.53*** (1.39, 1.68)
Being hospitalized for COVID		0.71** (0.59, 0.86)	0.71** (0.59, 0.86)
Childhood vaccination × Age			1.02* (1.00, 1.03)

Note: CI, confidence interval;

*** p < 0.001,

** p < 0.01,

* p < 0.05

**Table A3.** Sensitivity analysis

	Using mode and median imputation for missing data	Using the full set of confounders
Variables	OR (95%CI)	OR (95%CI)
Childhood vaccination (Ref. = No)	1.66*** (1.43, 1.94)	1.58*** (1.35, 1.85)
Schooling years	1.06*** (1.05,1.06)	1.06*** (1.05,1.07)
Age	1.03*** (1.03,1.03)	1.03*** (1.02,1.03)
Being married or partnered (Ref. = No)	1.52*** (1.44,1.60)	1.50*** (1.42,1.59)
Living in rural areas (Ref. = No)	0.80*** (0.76, 0.84)	0.83*** (0.79, 0.88)
Living in institutions (Ref. = No)	2.15*** (1.53, 3.03)	2.06*** (1.46, 2.92)
Self-rated health status (Ref. = Excellent)		
Very good	0.88 (0.77, 1.02)	0.99 (0.86, 1.15)
Good	0.76*** (0.67, 0.87)	1.05 (0.91, 1.20)
Fair	0.65*** (0.57, 0.74)	0.95 (0.82, 1.09)
Poor	0.41*** (0.35, 0.47)	0.62*** (0.53, 0.72)
Seeing children in person (Ref. = Daily)		
Several times a week	1.35*** (1.25, 1.46)	1.17*** (1.09, 1.26)
About once a week	1.49*** (1.38, 1.60)	1.25*** (1.15, 1.35)
Less often	1.25*** (1.17, 1.34)	1.09* (1.01, 1.17)
Never	0.99 (0.88, 1.11)	0.95 (0.85, 1.07)
Keeping social distance (Ref. = Never)		
Sometimes	1.22* (1.03, 1.44)	1.27** (1.07, 1.50)
Often	1.48*** (1.27, 1.72)	1.51*** (1.29, 1.76)
Always	1.69*** (1.46, 1.95)	1.68*** (1.44, 1.95)
Taking medication for COVID prevention (Ref. = No)	0.83*** (0.77, 0.89)	1.03 (0.96, 1.11)
Receiving flu vaccination (Ref. = No)	6.50*** (6.03, 6.99)	5.08*** (4.71, 5.48)
Ever tested positive for COVID-19 (Ref. = No)	0.70*** (0.62, 0.80)	0.74*** (0.64, 0.85)
Ever experienced any COVID symptoms (Ref. = No)	0.58*** (0.51, 0.66)	0.60*** (0.53, 0.68)
Forgoing medical care (Ref. = No)	1.48*** (1.39, 1.59)	1.42*** (1.32, 1.52)
COVID-19 lockdown stringency	1.02*** (1.02, 1.03)	1.00 (1.00, 1.01)
Losing a close person to COVID	1.53*** (1.39, 1.68)	1.44*** (1.31, 1.59)
Being hospitalized for COVID	0.71** (0.59, 0.86)	0.69** (0.56, 0.84)
Gender (Ref. = Women)		1.03 (0.98, 1.09)
Geographic regions (Ref. = Northern Europe)		
Southern Europe		0.29*** (0.24, 0.35)
Eastern Europe		0.13*** (0.11, 0.16)
Western Europe		0.36*** (0.30, 0.44)
Israel		0.55*** (0.40, 0.75)
Frequency of contacting children online		
Online (Ref. = Daily)		
Several times a week		1.06 (0.99, 1.12)
About once a week,		1.17*** (1.07, 1.28)
Less often		1.08 (0.97, 1.20)
Never		0.91 (0.80, 1.03)

Note: CI, confidence interval;

*** p < 0.001,

** p < 0.01,

* p < 0.05
